# Non‐freezing cold injury: A little‐known big problem

**DOI:** 10.1113/EP091139

**Published:** 2023-02-20

**Authors:** Mike Tipton, Clare Eglin

**Affiliations:** ^1^ Extreme Environments Laboratory SHES, University of Portsmouth Portsmouth England

Non‐freezing cold injury (NFCI) is an enigma. Very few people have heard of this thermal injury to the hands and feet, but in one form or another it has caused widespread and significant problems for centuries. It has influenced the outcome of military campaigns including, nearly, the Falklands campaign (Golden et al., 2013). In addition, it is associated with significant compensation claims against the UK MoD, averaging £23.5 million per annum between 2016 and 2020 (https://www.gov.uk/government/statistics/mod‐common‐law‐compensation‐claims‐statistics‐202122/mod‐common‐law‐compensation‐claims‐statistics‐202122). It is a major hazard for those venturing into cold, wet environments, as well as those sleeping rough or engaging in the new craze of open, cold water swimming (Tipton et al., 2017). It can result in lifelong debilitation including persistent pain, hyperhidrosis and cold sensitivity, in extreme circumstances it may necessitate amputation of the injured extremity.

It has appeared in various guises over the years, ‘trench foot’, ‘immersion foot’ and ‘cold immersion injury’. Yet, despite its prevalence, impact and long history, little is known about its pathogenesis and pathology – we need to know more. We know that exposure to cold and wet conditions can result in NFCI, but have no idea of the exact dose required to cause it. Although factors that predispose to NFCI have been identified (Burgess & MacFarlane, 2009; Golden et al., 2013; Kuht et al., 2019), none apply in all conditions and, even with similar cold exposures, it remains impossible to accurately predict those who will suffer severe NFCI as opposed to those who will remain uninjured.

Even more frustratingly, we remain unsure of the mechanisms causing the injury. Its pathology has not been definitively described, which makes screening, assessing, preventing and treating NFCI challenging. One reason for this lack of clarity is down to the way we do science: because the symptoms of NFCI include neural (possibly small fibre neuropathy) and vascular (possibly endothelial microvascular damage) components, groups specialising in these aspects have independently investigated NFCI. But NFCI itself could be a neurovascular injury with a primary nerve injury affecting vascular function, or a vascular injury causing nerve damage, or purely neural in some cases and vascular in others. Different individuals may be susceptible to either or both pathologies. Alternatively, the type of cold exposure (e.g., intensity, duration, wettedness, static/dynamic, etc.), coincident factors (e.g., stress, dehydration, deep body temperature, age, sex hormones, genetic predisposition) and comorbidities (e.g., peripheral vascular disease) may result in one form of NFCI pathology or another (Tipton et al., 2020)

It follows that a multidisciplinary approach to try to unravel the pathology of the symptoms we term NFCI has more prospect of success than the examination of one feature, be that neural or vascular. This, like most big problems, requires collaboration, and in this edition of *Experimental Physiology* there are three back to back papers on the topic of NFCI that are the first to examine the condition in one investigation from both the neural and vascular perspectives.

The three studies compared the responses of individuals with mild to moderate chronic NFCI to matched controls with (COLD) or without (CON) similar previous cold exposure, thus addressing some of the criticisms of earlier research studies. The inclusion of the COLD group enabled examination of the effect of cold exposure per se in the absence of cold injury. Also, ethnicity is known to be a risk factor for NFCI and therefore the control groups were closely matched in this and other respects. Individuals with chronic NFCI were found to be more cold sensitive than the control groups. Vascular tests (post‐occlusive reactive hyperaemia, local heating iontophoresis of acetylcholine and sodium nitroprusside and deep inspiration) indicated that this was not due to an impairment in endothelial function or an augmented sympathetic vasoconstrictor response (Eglin, Wright, Maley, et al., 2023).

Quantitative sensory testing indicated that individuals with chronic NFCI were hyposensitive to mechanical and warm stimuli, possibly due to a reduction in intraepidermal nerve fibre density (Wright et al., 2023). Measurement of blood biomarkers indicated that mild to moderate chronic NFCI is not associated with either oxidative stress or a pro‐inflammatory state (Eglin, Wright, Shepherd, et al., 2023). Plasma interleukin‐10 and syndecan‐1 concentrations were elevated in the NFCI and COLD group indicating an effect of cold exposure per se (Eglin, Wright, Shepherd, et al., 2023).

Interindividual variation was observed in all of the groups for every variable measured, making a clinical diagnostic cut‐off for NFCI problematic and underlining the difference between statistical difference and applicable clinical criteria for potentially ending a career. The greatest discriminator was the perception of cold and discomfort in the area with chronic NFCI, despite similar skin temperatures to the control groups (Eglin, Wright, Maley, et al., 2023), potentially due to elevated endothelin‐1 concentration (Eglin, Wright, Shepherd, et al., 2023). For a variety of reasons, objective rather than subjective criteria of NFCI would be preferable.

It is evident that further research is required to verify the findings presented here; a longitudinal study with appropriate control groups is desperately needed to follow the progression of NFCI from the initial injury to resolution. Any future studies into NFCI must be multidisciplinary and also consider the biopsychosocial impact of the pain associated with chronic NFCI.

## AUTHOR CONTRIBUTIONS

Both authors approved the final version of the manuscript and agree to be accountable for all aspects of the work in ensuring that questions relating to the accuracy or integrity of any part of the work are appropriately investigated and resolved. All persons designated as authors qualify for authorship, and all those who qualify for authorship are listed.

## CONFLICT OF INTEREST

The authors confirm that neither author has any conflict of interest.

## FUNDING INFORMATION

No funding received for this work.
